# A New Effort to Diversify Faculty: Postdoc-to-Tenure Track Conversion Models

**DOI:** 10.3389/fpsyg.2021.733995

**Published:** 2021-11-05

**Authors:** Dawn Culpepper, Autumn M. Reed, Blessing Enekwe, Wendy Carter-Veale, William R. LaCourse, Patrice McDermott, Robin H. Cresiski

**Affiliations:** ^1^University of Maryland, College Park, College Park, MD, United States; ^2^University of Maryland, Baltimore County, Baltimore, MD, United States

**Keywords:** postdoc, diversity, faculty diversity, higher education, AGEP

## Abstract

Calls to diversify the professoriate have been ongoing for decades. However, despite increasing numbers of scholars from underrepresented racial minority groups earning doctorates, actual progress in transitioning to faculty has been slow, particularly across STEM disciplines. In recent years, new efforts have emerged to recruit faculty members from underrepresented racial minority groups (i.e., African American/Black, Hispanic/Latinx, and/or Native American/Native Hawaiian/Indigenous) through highly competitive postdoctoral programs that allow fellows the opportunity to transition (or “convert”) into tenure-track roles. These programs hybridize some conventional aspects of the faculty search process (e.g., structured interview processes that facilitate unit buy-in) along with novel evidence-based practices and structural supports (e.g., proactive recruitment, cohort communities, search waivers, professional development, enhanced mentorship, financial incentives). In this policy and practice review, we describe and synthesize key attributes of existing conversion programs at institutional, consortium, and system levels. We discuss commonalities and unique features across models (*N* = 38) and draw specific insights from postdoctoral conversion models developed within and across institutions in the University System of Maryland (USM). In particular, experience garnered from a 10-year-old postdoc conversion program at UMBC will be highlighted, as well as the development of an additional institutional model aimed at the life sciences, and a state-system model of faculty diversification with support from a NSF Alliances for Graduate Education and the Professoriate (AGEP) grant.

## Introduction

Despite recent demographic shifts in undergraduate college student enrollment and concerted federal, institutional, and foundation efforts, the percentage of faculty members who come from underrepresented racial minority groups^1^ in tenured and tenure-track positions remains small, particularly in STEM fields (Griffin, 2020; Smith, 2020). Interventions in this area often focus on increasing the number of underrepresented racial minority scholars interested in and prepared for faculty careers or providing institutional incentives (“target of opportunity” hiring programs) for the hiring of faculty from underrepresented minority groups (Griffin, 2020). Yet, structural barriers continue to play a significant role in the persistence of faculty racial gaps. Racial bias (Eaton et al., 2020; White-Lewis, 2020), hostile climate (Zambrana, 2018), narrow conceptions of scholarly excellence and quality (Hoppe et al., 2019; Settles et al., 2020), and workload inequities (Jimenez et al., 2019; Misra et al., 2021) cumulatively undercut diversity efforts focused on recruitment and hiring alone. Increasing the diversity of the faculty therefore requires strategic and systemic interventions, focusing on recruitment and retention but also organizational transformation and change (Griffin, 2020; Smith, 2020).

One emerging, yet understudied, intervention in this area are postdoctoral fellowship programs that seek to “convert” postdocs to faculty positions within the institution or systems in which they complete their fellowship. Such “grow your own” programs subvert norms of traditional postdoctoral programs, wherein postdocs typically work directly on the research of a single faculty member and then find a faculty position elsewhere (Griffin, 2020; Flaherty, 2021). Conversion programs have the potential to directly increase faculty diversity by (a) creating a talented pool of qualified fellows; (b) intentionally recruiting postdocs to departments/institutions with an interest in hiring them; and (c) creating internal commitments within departments and institutions to the professional success of scholars from underrepresented racial groups (Flaherty, 2021). Yet, because conversion programs are relatively new, the field lacks an understanding of the processes, practices, and policies that have been used to create, institutionalize, and sustain these new models.

The goal of this Practice and Policy Review is to fill that gap. We draw from an online, preliminary review of postdoctoral conversion programs aimed at increasing faculty diversity and our own experiences in designing, implementing, and evaluating postdoctoral conversion programs on our campuses in the University System of Maryland (USM). Based on these experiences and data, we suggest a five-stage model that institutions could consider in establishing their own postdoctoral conversion program(s) for faculty diversity. This model is intended to be the basis for future research, replication, and adaption.

This topic merits study and is timely for several reasons. For years, students have demanded that institutions hire, more faculty members from underrepresented racial minority groups, and these demands have intensified in the context of the protests for racial justice in 2020 and 2021 (Kezar and Fries-Britt, 2018; Ezarik, 2021). Postdoc conversion models therefore offer one way for institutions to fulfill their equity goals. As such, this study focuses on challenging institutions to think through the steps they can take to mitigate structural barriers to the professoriate for historically marginalized faculty through postdoc conversion models.

The structure of this review is as follows. First, we discuss the current ethno-racial demographics of academics in the United States and examine why interventions at the postdoctoral level are needed. Next, we describe our methods and how we arrived at our five-stage postdoctoral conversion model. Then, we discuss each stage of the model and make actionable recommendations, drawing examples from our own experiences and our program review. Finally, we discuss our findings and make suggestions for areas of future study.

### Faculty Demographics in the United States

Table 1 shows the racial distribution of the U.S. population based on the most recent Census in 2020 compared to the racial distribution of graduate students, postdoctoral appointees, and faculty members in U.S. higher education institutions. When we compare the racial makeup of faculty in the last column, we see that White faculty members compose 73.15% of all faculty members, which closely represents their 76.3% share in the Census. However, the composition of faculty members from racially minoritized groups is not representative of their respective share of the population. For example, Asian faculty members make up 10.83% of all faculty, almost twice that of their makeup in the population (5.9%). Scholars who identify as Black/African American (5.84% vs. 13.4%), Hispanic/Latino (10.83% vs. 18.5%), or Native American (0.44% vs. 1.3%) are underrepresented in the faculty compared to their percentage share in the population.

**TABLE 1:** Percent distribution of graduate students, postdocs and faculty by race and ethnicity.

Ethnicity and race	US population	Doctoral students (all disciplines)^*^	Postdocs (science and engineering)^1^	Faculty (all disciplines)^2^
Hispanic/Latino	18.5%	8.07%	6.53%	5.29%
Non-Hispanic/Latino				
*American Indian or Alaska Native*	1.3%	0.34%	0.23%	0.44%
*Asian*	5.9%	9.70%	20.00%	10.83%
*Black or African American*	13.4%	7.12%	3.69%	5.84%
*Native Hawaiian/Pacific Islander*	0.2%	N/A%	0.18%	0.16%
*White*	76.3%	68.74%	57.63%	73.15%
*Two or More Races*	2.8%	3.18%	1.76%	1.07%
Unknown Ethnicity and Race	–	2.84%	9.97%	3.22%

*National Science Foundation Survey of Earned Doctorates, 2019, table 19: https://ncses.nsf.gov/pubs/nsf21308/data-tables.

*^1^ National Science Foundation Survey of Graduate Students and Postdoctorates in Science and Engineering, Fall 2019, table 2-1: https://ncses.nsf.gov/pubs/nsf21318#data-tables.*

*^2^ National Center for Education Statistics, 2018: https://nces.ed.gov/programs/digest/d19/tables/dt19_315.20.asp.*

Table 1 also illustrates that as levels of training increase, the percentage of underrepresented racial minority scholars decreases. Data from the 2019 National Science Foundation Survey of Graduate Students and Postdoctorates in Science and Engineering^2^ indicates that among U.S. citizens and permanent residents, most pre-professoriate scholars are White. However, there is relatively greater diversity among underrepresented racial minority doctoral students and postdocs compared to faculty. This suggests that the postdoc to faculty transition is a critical juncture at which interventions should focus (Gibbs et al., 2015; Meyers et al., 2018).

### Postdoctoral Faculty Diversity Programs

An emerging body of research shows that postdocs from underrepresented racial minority groups encounter numerous challenges as they navigate their fellowships. Factors such as racial bias and stereotypes, inadequate mentoring, poor job market prospects, and competitive and hostile cultures lead to waning interest in academic careers among underrepresented racial minority postdocs scholars (Gibbs et al., 2015; Jaeger and Dinin, 2017; Lambert et al., 2020). While such studies point to specific structures (e.g., mentoring) that need to be altered to enhance postdoc retention, relatively few studies examine integrated postdoc training models and how they might be linked to the successful transition of underrepresented minority postdocs into faculty careers within the institutions that host them. For example, a handful of studies indicate that access to multiple mentors and multi-institutional training (e.g., across institutions with different missions) can be beneficial in preparing underrepresented racial minority postdocs for faculty careers (Holtzclaw et al., 2005; Faupel-Badger and Miklos, 2016; Eisen and Eaton, 2017). However, in most of these programs, the goal is that the postdoctoral fellow completes their fellowship and then takes a faculty position at another institution. That is, most diversity postdoc programs are not intended to directly increase faculty diversity at the institution at which the fellow is trained.

These models are based upon long-standing norms within postdoctoral training, but they can also present tensions. Institutions may devote significant resources to the creation of a diversity postdoc program that results in a short-term “boost” to diversity, but this boost is not sustained after the fellow completes their term. External grants (e.g., from the National Science Foundation or National Institutes of Health) have catalyzed many postdoc diversity programs, which then end upon the grant’s completion. There may be a misalignment between the fellow’s expectations about the prospect being hired into the department and the department’s ability to hire, which can breed resentment and ill-will. These postdoc diversity initiatives, in isolation, may be insufficient to move the needle significantly (Meyers et al., 2018).

This is where our, the authors, experiences come into play. Six of the authors have been directly involved with the development and implementation of postdoctoral conversion programs that are responsive to individual postdocs needs and the structural barriers that can encumber successful transition to the tenure-track. We have all also been involved in national and disciplinary-based conferences on postdoctoral training and its potential to contribute to faculty diversity. As a collective, we have been involved with:

The University of Maryland, Baltimore County (UMBC) Postdoctoral Fellowship for Faculty Diversity, which is now in its tenth year. This program has hosted 20 scholars, and of those who have completed the program, has converted 11 of 20 underrepresented postdocs into tenure-track lines at UMBC (7 of 20 are tenure-track faculty nationally), one of whom has just received tenure. The UMBC College of Natural and Mathematical Sciences Pre-Professoriate Program (PFP), which has converted all of its participants to tenure-track positions at UMBC;

A modified President’s Postdoctoral Fellowship Program at the University of Maryland, College Park (UMCP)^3^

An NSF-funded AGEP PROMISE Academy, a state system-wide postdoc conversion model to diversify biomedical faculty, under development via a consortium of five USM institutions (Salisbury University; Towson University; University of Maryland Baltimore; UMBC; UMCP). The AGEP PROMISE Academy state system model is fleshed out in a case report in this issue (Cresiski et al., submitted^4^).

With these experiences in view, we see the promise and potential limitations of postdoc conversion programs for enhancing faculty diversity. We thus undertook this review as a way to bring together our collective insights and data from the field to propose a conversion model.

## Policies and Practices in Postdoctoral Conversion

We took an integrative approach to considering the postdoc conversion policies and practices. Specifically, we drew from a review of postdoctoral conversion programs (Table 2) as well as our own experience in creating, administering, and evaluating postdoctoral conversion programs on our own campuses and within our university system.

**TABLE 2:** Postdoc-to-tenure track conversion programs in United States.

Institution	Program name
(1) Binghamton University	Presidential Diversity Postdoctoral Fellowship
(2) Broad Institute of MIT and Harvard	Postdoctoral Research Opportunity Diversity Initiative
(3) Carleton College	Oden Postdoctoral Fellows
(4) Carnegie Mellon University^1^	President’s Postdoctoral Fellowship Program
(5) Emory University School of Medicine	FIRST A Postdoctoral Fellowship Program at Emory
(6) Georgia Tech University^1^	President’s Postdoc Program
(7) Harvard University	Mary Fieser Postdoctoral Program for Women and Minorities (2008–2013)
(8) Johns Hopkins University	Provost’s Postdoctoral Fellowship Program
(9) Miami University	Instructor/Visiting Assistant Professor and Heanon Wilkins Fellow
(10) New York University^1^	Provost’s Postdoctoral Fellowship Program
(11) Northeastern University	STEM Future Faculty Postdoctoral Fellowship Program
(12) Ohio State University	Dean’s Diversity Postdoctoral Fellows
(13) Syracuse University	Chancellor’s Faculty Fellowship
(14) University of California	President’s Postdoctoral Fellowship
(15) University of California, Berkeley^*^	Chancellor’s Postdoctoral Fellowship
(16) University of California, Davis^*^	Chancellor’s Postdoctoral Fellowship Program
(17) University of California, Irvine^*^	Chancellor’s ADVANCE Postdoctoral Fellowship
(18) University of California, Los Angeles^*^	Chancellor’s Postdoctoral Fellowship Program
(19) University of California, Merced^*^	Chancellor’s Postdoctoral Fellowship Program
(20) University of California, Riverside^*^	Chancellor’s Postdoctoral Fellowship
(21) University of California, San Diego^*^	Chancellor’s Postdoctoral Fellowship Program
(22) University of Chicago	Provost’s Career Enhancement Postdoctoral Fellowship
(23) University of Illinois at Chicago	Bridge to the Faculty
(24) University of Colorado, Boulder^1^	Postdoctoral Fellowship Program for Academic Diversity
(25) University of Iowa	Provost’s Postdoctoral Faculty Fellowship Program
(26) University of Maryland, Baltimore County	Pre-Professoriate Fellowship in Biological Sciences
(27) University of Maryland, Baltimore County	Postdoctoral Fellows Program for Faculty Diversity
(28) University of Maryland, College Park^1^	President’s Postdoctoral Fellowship Program (part of FAMILE: Faculty Advancement at Maryland for Inclusive Learning and Excellence)
(29) University of Michigan^1^	President’s Postdoctoral Fellowship Program
(30) University of Minnesota^1^	President’s Postdoctoral Fellowship Program
(31) University of New Hampshire	Postdoctoral Diversity and Innovation Scholars program
(32) University of New Mexico	University of New Mexico’s Inclusive Excellence Post-Doctoral and Visiting Scholars Program (IEPDVSP)
(33) University of North Carolina at Charlotte^1^	Multicultural Postdoctoral Fellowship Program
(34) University of North Carolina Chapel Hill^1^	The Carolina Postdoctoral Fellowship for Faculty Diversity
(35) University of Rhode Island	Multicultural Postdoctoral Fellowship
(36) University of Wisconsin – Madison	Anna Julia Cooper Postdoctoral Fellowship
(37) Vanderbilt University	Academic Pathways Program
(38) Wayne State University	Postdoctoral to Faculty Transition Fellowship Program

^*^Part of the University of California President’s Postdoctoral Fellowship Program.

*^1^Part of the Partnership for Faculty Diversity at the University of California.*

### Methods

Our first step was to generate a list of postdoc diversity programs to include in our review. We focused on postdoc programs that:

(a) explicitly focused on increasing faculty diversity (i.e., excluded programs that did not specify diversity as a goal); and
(b) specifically mentioned conversion or transition to the tenure-track at the host institution as a possibility or goal for postdocs that participated.

To generate a list of programs to include, we first reviewed the diversity postdoctoral programs listed on minoritypostdocs.com, a website dedicated to career development and resources for scholars of color. We added to that initial list postdoc programs we were aware of based on our own networks and experiences (e.g., AGEP programs or institutions with programs not listed, which we generated from a Google search of “postdoc diversity programs”). Next, we reviewed the program websites of each program, determining which programs met the criteria above. Based on this, we narrowed the list to 38 postdoctoral conversion programs across the country (Table 2). For each program on this list, we noted the policies (e.g., search waivers) and practices (e.g., mentor training; annual reviews) specified on their websites that seemed to align with the goal of conversion.

Finally, we considered how these policies and practices mapped on to our own experiences in developing, implementing, and managing postdoctoral conversion programs on our own campuses and how policies and practices might best fit together or be sequenced to further the goals of increasing faculty diversity. For example, although some programs may not specify the process of conversion to the tenure-track, we recommended, based on our own experiences, that these expectations are made clear during recruitment. In this way, we identified discrete periods of time and activity based on how programs in our review sequenced various aspects of their models, as well as our own insights in what has worked (or needs to be improved).

We organized our findings into five stages: (1) Laying the Foundation; (2) Recruiting Fellows, Matching to a Mentor/Department and Pre-Arrival Preparation; (3) Fellowship Period; (4) Conversion to the Tenure-Track; and (5) Ongoing, Iterative Evaluation for Program Improvement. We consider these to be the five stages institutions, systems, or consortiums might follow to create a postdoc conversion program with the goal of increasing faculty diversity. We discuss policies and programs relevant to each stage and draw specific examples from our review of national postdoc conversion programs as well as practical experience within the USM.

A discussion of limitations is warranted. Although we attempted to capture the breadth of postdoctoral conversion programs across the country, we did so based on a convenient sample of programs websites that could be accessed publicly. These data are incomplete in many ways, including the possibility that there may be postdoc conversion programs not included in this review; and that institutions may in reality use some of the practices (e.g., a search waiver) even if such information was not available on their website. For these reasons, we do not present evidence on the number of institutions that adopted certain practices (e.g., 10/38 had a mentoring program) because the data would not be conclusive. Moreover, as we have learned through our experiences, creating a sustainable postdoc conversion model is an iterative and non-linear process. Institutions may wish to alter the sequence of stages or place different policies or practices into different stages. While our goal is to offer common policies and practices for consideration and potential adaptation, we nevertheless acknowledge these as limitations to our approach. Ultimately, we hope that this discussion will spur additional scholarly literature and institutional transparency on this topic.

### STAGE 1: Laying the Foundation

Stage 1 encompasses the foundational work required before beginning a program. This stage encourages institutions to honestly assess where they currently stand with the diversity of its faculty. It involves an institution critically examining pre-existing programs, identifying structural barriers, and looking to practices at other institutions (as we strive to accomplish in this report). Stakeholders must also decide how a postdoc conversion program will be funded and build and secure financial and operational commitments at multiple levels, including the department, college, and executive leadership of the institution. And perhaps most importantly, institutions must determine who will execute and lead the program.

#### Assessing Existing Faculty Diversity Efforts

One of the most critical elements in establishing a postdoc conversion program is for the institution to place it within the context of existing faculty diversity efforts and extant practices. For example, after years of insufficient progress with the recruitment and retention of faculty from underrepresented racial minority groups, in 2010, UMBC established the Executive Committee on the Recruitment, Retention, and Advancement of Underrepresented Faculty (henceforth called “Executive Committee”), a group of tenured faculty members of color, co-chaired by the provost and one of the committee members, to lead UMBC’s faculty diversity efforts. This group did an analysis of existing initiatives and efforts on campus, including the UMBC’s NSF-ADVANCE Program and existing AGEP programs focused on graduate education, as well as an analysis of faculty diversity programs at other institutions across the country. The Executive Committee determined that previous diversity hiring practices such as incentive hiring and target-of-opportunity hiring were unsuccessful because they failed to address the underlying issues of inhospitable departmental climates, bias, and institutional racism. This group then identified UNC Chapel Hill’s Postdoctoral Fellowship for Faculty Diversity program and the University of California System (UC System) President’s Postdoctoral Fellowship Program as relevant and successful models to emulate. There is some limited evidence that other postdoc conversion programs have likewise dovetailed on existing faculty diversity efforts. For instance, the Northeastern Future Faculty Fellowship Program and Syracuse University’s Chancellor’s Faculty Fellowship explicitly state that their programs emerged in relation to ADVANCE programs.

Understanding relevant local, state, and national employment regulations is also critical at this point. For instance, as UMBC program leaders designed the Fellowship for Faculty Diversity program, some prominent faculty questioned the constitutional and statutory legality of the program, more specifically the focus on scholars from underrepresented racial minority groups. Program leaders were able to cite the existence of National Science Foundation programs and models like UNC Chapel Hill’s Postdoctoral Fellowship for Faculty Diversity and the UC System’s President’s Postdoctoral Fellowship Program to legitimize the program goals, therefore mitigating some resistance.

#### Establishing Structure and Co-leadership

Another critical piece is for institutions to establish program and leadership or co-leadership structure. Many of the postdoctoral programs reviewed seem to be managed and facilitated centrally by the provost’s office or faculty affairs office. For example, postdoc conversion programs at John Hopkins University and Northeastern University are centrally managed by faculty affairs offices within academic affairs. The UMBC Fellowship for Faculty Diversity Program uses a co-leadership model: unlike many other programs, UMBC’s Executive Committee is the main advisory body for the program, putting genuine authority in the hands of faculty of color, though the fellows are funded (and the application process is managed) by faculty affairs. Similarly, UMCP’s program is funded by faculty affairs, reviewed by a diverse committee of university faculty, and the application process is managed by the office of postdoc affairs. To operate a system-wide program, the UC System’s President’s Postdoctoral Fellowship Program has central administrators who are employed by the system. Similarly, the USM AGEP PROMISE Academy is administered by a leadership team composed of graduate deans, faculty affairs administrators and postdoctoral affairs staff from across the five-institution alliance. While a part-time director ensures continuity and accountability, the co-leadership from participating institutions creates meaningful buy-in that supersedes silos and potential power dynamics.

There are also examples of conversion models housed within academic colleges, including UMBC’s Pre-Professoriate Program, which is located within the College of Natural and Mathematical Sciences, and The Ohio State University’s (OSU) Dean’s Diversity Postdoctoral Fellows in the College of Education and Human Ecology. In the case of the Pre-Professoriate Program, the decision to have a college-level program was a strategic one. Given the expectations and resources needed to prepare scholars for tenure-track roles in the life sciences (including laboratory space and startup funds), program designers created a program parallel yet distinct from the centrally managed Fellowship for Faculty Diversity Program. The dean’s office manages the Pre-Professoriate Program, and it has its own requirements and expectations.

#### Creating Application Processes, Procedures, and Cost-Sharing

Based on our review, most postdoc conversion programs outline a competitive process, wherein candidates apply centrally, departments put forward candidate application packages they determine to be a good match, and a central academic administrator or committee (e.g., provost’s office; a committee; a dean) determines which departments/units will be granted a postdoc position. The mechanisms by which applications are generated and put forward vary substantially. For example, in the UMBC Fellowship for Faculty Diversity, departments review applicants and submit their own requests to their dean. The dean then makes recommendations to the Executive Committee, who selects finalists for interview. After interviews with the departments and a variety of stakeholder offices, the Executive Committee decides which candidates to offer to the positions. These assessments are based on factors such as candidate qualifications, availability of appropriate mentors, and departmental readiness to retain and support the advancement of underrepresented scholars (see Stage 2). Similar competitive processes are in place at institutions like the University of Illinois at Chicago, UNC Chapel Hill, and Northeastern University, or in place at the unit level such as in UMBC’s Pre-Professoriate Program or OSU’s Dean’s Diversity Postdoctoral Fellows Program. To maximize departmental faculty buy-in, the Pre-Professoriate program adopted a standard faculty search process to hire each Pre-Professoriate fellow. Institutions participating in the President’s and Chancellor’s Postdoctoral Fellowship Programs (including UMCP) use a centralized application system (i.e., fellows could apply centrally to be postdocs at more than one institution). However, even with a centralized application mechanism, there are institutional processes that must be determined (for example, a department may write letters of support for selected candidates and forward applications to the dean or the faculty review committee, which reviews the materials and makes recommendations to the provost, etc.).

In terms of funding the initial postdoc period, we found significant variability. Several programs (e.g., UMCP), specified a cost-sharing structure where the initial postdoc salary/stipend is shared between central academic affairs and the host department. Other institutions, such as Johns Hopkins University, Northeastern University, and the University of Chicago Illinois, appear to offer full central funding (e.g., from academic affairs) during the fellowship period, with varying levels of central salary subsidy after the postdoc converts (see Stage 4). Likewise, there is variation in how postdoc resources (e.g., professional development, research funds) are funded. We see benefits in either approach. One on hand, cost-sharing strategies may enhance departmental buy-in and ensure that departments recruit only those candidates that they think will be successful. On the other hand, fully subsidized postdoc salaries may incentivize departments with fewer resources to participate. In the AGEP PROMISE Academy Alliance, the five institutions within the alliance must determine mechanisms to fund the fellows, but professional development, travel, and some research funds are covered by the NSF grant.

#### Classification of Postdocs and Joint Titling

Early-on, it is imperative that institutions engage with Human Resources to determine how the postdocs will be classified. Postdoctoral positions are not uniformly standardized at or across institutions in the U.S., a fact that has made research about this population notoriously difficult (McConnell et al., 2018). Titles and classifications directly impact a fellow’s access to institutional and departmental resources, how they are perceived by colleagues, and the hiring or conversion process itself. For conversion, it is important to distinguish whether postdocs are university faculty members or trainees. This difference in employment status may mean that some postdocs receive a salary while others receive stipends, which requires different tax treatments. Although all postdocs have access to health and dental insurance through the university, payment and withholding arrangements differ.

One option employed by some institutions is joint titling, offering a faculty position/title concurrently to the postdoctoral one. The University of Chicago’s Provost’s Career Enhancement Postdoctoral Fellowship appoints fellows under a classification of “Instructors on the tenure track” with the intent that they will be promoted to Assistant Professor at the end of the fellowship period. UMBC’s Pre-Professoriate program hires fellows into Research Assistant Professor roles, a non-tenure track faculty rank, that gives scholars all the benefits of being classified as faculty and acknowledges the mutual intentions of the fellow and the department to have the fellow become a tenure-track faculty member.

### STAGE 2: Recruiting Fellows, Matching Fellows to a Mentor/Department and Pre-arrival Preparation

Stage 2 focuses on recruiting fellows, identifying faculty mentors and host departments, establishing and communicating expectations, hiring fellows, and preparing for their arrival. Programs that seek to garner a large pool of applicants should begin their recruitment process by creating an active recruitment plan as well as actively engage departments in recruitment. Steps should also be taken to ensure postdocs, proposed faculty mentors, and departments understand the intent, structure, and their responsibilities within the program.

#### Recruiting Fellows

In creating a recruitment plan, programs should generally engage in assessing policies and procedures for recruiting and hiring fellows to make sure a robust, evidence-based plan can be created. For example, prior to beginning recruitment for all UMBC’s Fellowship for Faculty Diversity fellows (and indeed for all faculty positions), the Executive Committee requires each department to develop a comprehensive ‘faculty diversity hiring and recruitment plan’ that includes a discussion of search committee composition, an active recruitment strategy, inclusive draft job advertisement, and initial evaluation and interview strategy. The Dean’s Office and Provost Office review these plans before searches are authorized. Additionally, UMBC implemented *Interfolio: Faculty Search*, an online software, which increased the transparency of the faculty search committee’s candidate review and provided a tool to track the diversity of the applicant pools. A webpage for the program was also created to provide information to potential applicants. In addition, leaders can also look at the national pipeline of doctoral degrees by discipline based on the annual Survey of Earned Doctorates (SED) and compare this to faculty applicant pools, finalist pools, and hires within departments across the institution. This data assessment provides an opportunity for discussion to move beyond anecdotal evidence^5^.

After institutions put internal procedures into place, they must engage in well-documented recruitment approaches to increase the pool of underrepresented applicants (Peek et al., 2013; Bhalla, 2019). The primary method is centered on utilizing existing networks, through national associations, and through regional and national conferences. Regional and national conferences that focus on retainment of minoritized communities such as, the Southern Regional Education Board (SREB), the Society for the Advancement of Chicanos and Native Americans in Science (SACNAS), the National Society for Black Engineers (NSBE) are common avenues for postdoctoral program recruitment. In the case of UMBC’s Fellowship for Faculty Diversity, the Executive Committee also relied on “The Committee on Strategies and Tactics to Recruit to Improve Diversity and Excellence” (STRIDE), a program in which respected faculty members support the efforts of search committees, departments/programs, and colleges to recruit, retain, and promote diverse faculty and foster more inclusive and equitable academic spaces for faculty peers. Likewise, the AGEP PROMISE Academy developed a Guidance Document for the Recruitment of AGEP PROMISE Academy Fellows^6^ to ensure semi-standardized practices that leverage evidence-based approaches in the recruitment of fellows across the alliance institutions. This document includes sample job advertisement language, appendices of minority graduate and postdoctoral directories, email addresses of top minority Ph.D. producing programs in the biomedical sciences, and sample rubrics for the evaluation of candidates. The overall goal of such practices is to give departments tools for being more proactive in recruiting potential postdocs.

#### Creating a Mentor/Departmental Match Process

Faculty diversity programs around the country emphasize the fundamental role of effective mentorship during the fellowship period. In a departure from the traditional model of fellows supporting their mentors’ research, successful postdoctoral conversion programs aim to support fellows’ independent research and teaching. This component is clearly outlined by such programs such as those in the UC System, the University of Michigan, and the UNC Chapel Hill, and programs in USM. Across these programs, we observed relative consensus that appropriate mentors would be those who had an established track record of mentoring and were tenured faculty members, although a handful of programs indicated that untenured faculty may be “involved” as mentors (though not the primary mentor).

The process by which mentors and corresponding departments are identified varies. In some models, such as UMBC’s Fellowship for Faculty Diversity, fellows apply to the program centrally at the institution or college level. Once departments receive a candidate’s application and determine the criteria by which the postdoc will be evaluated, they internally identify willing and appropriate mentors. This approach ensures that a fellow’s application is assessed based on the strength of their skills and alignment with department needs, rather than putting the onus on the fellow to identify departments and mentors in which there may be a fit. If departments are interested in supporting a postdoc, they then submit a detailed mentoring plan as part of the overall application process. In fact, a few programs including UMBC’s Fellowship for Faculty Diversity program and the University of Rhode Island’s Multicultural Postdoctoral Fellowship in the Biological Sciences, require departments to identify a primary faculty mentor and a secondary mentor outside of the fellow’s program as part of their application process.

Other programs require the applicant to identify a proposed mentor and department in their application. For example, most of the Presidential and Chancellor Postdoctoral Fellows Programs, including UMCP’s, require applicants to solicit and subsequently submit a letter from the proposed mentor, department chair, and sometimes the dean, that indicates their support for the postdoc. This method ensures that the pool of potential postdocs is composed of candidates who already have faculty and department support.

One aspect that was unclear from our review was when, if, and how candidates were interviewed and by whom. Traditional postdocs are often interviewed only by the hiring faculty member if interviewed at all. But postdocs that are going to be considered potential faculty colleagues require a different degree of vetting by a broader set of stakeholders. For UMBC’s two conversion programs, extensive interviewing is done with the potential hiring department, department chair, and deans among others, similar to that of a traditional, national faculty search. For the AGEP PROMISE Academy fellowship, leadership aims to set up research talks and networking events with fellows and institutions of interest as informal interviews to assist fellows in connecting with departments that are potential hiring departments within the university system. An interesting pilot program, the Cottrell Emerging Scholars Program^7^, facilitates underrepresented postdoctoral candidates (from programs which do not include conversion, like Vanderbilt’s Academic Pathways^8^ program) to visit other campuses within a consortium for an intensive mock faculty job interview. This clearly serves as an opportunity for professional development for the postdoc, but it also has become a recruitment mechanism for the departments hosting the mock interviews and has directly led to placement of fellows in tenure track positions. While this is not yet a full-fledged postdoc conversion model, aspects of this program may be worthy of replication especially for those considering consortia or system approaches.

One emerging practice we found at a few institutions was the creation of mentor development programs. For example, at UMCP, faculty members who are the mentors of President’s Postdoctoral Fellows are required to participate in mentoring training that uses the Entering Mentoring framework^9^. We are also aware of mentoring trainings that occur in the Big 10 Academic Alliance as part of their AGEP programs^10^. While all programs in our review specified a mentoring component, professional development/training for mentors was not universally required.

#### Assessing Readiness

As discussed, many postdoc conversion programs are competitive processes, wherein departments submit applications and a central hiring authority (e.g., dean, provost, committee) decides which units will be granted funds to host a postdoc. Our review uncovered several criteria by which these determinations are made, including readiness assessments and future hiring needs.

An emerging practice in this area is an assessment of “departmental readiness” to welcome, support, retain, and help advance scholars of color. Some institutions, like UMBC, have put in place mechanisms to examine if departments applying to have a postdoc have environments that are inclusive, welcoming, and are places where a scholar is likely to be retained. Determination about a department’s readiness is made based on evaluating the quality of the mentoring or retention plans the department submits with the application package; examining the department’s history of recruiting, retaining, and mentoring faculty from underrepresented racial minority groups; and/or participation in relevant diversity-related assessments or trainings. Other institutions, such as UMCP, mention the evaluation of a retention plan, though the details of the plan are not specified. Similarly, other institutions mention mentoring plans as required, but it is not clear if plans are reviewed/assessed as part of the postdoc award process.

Another criterion by which readiness might be evaluated is the extent to which the department will be able to hire when the postdoc’s fellowship is complete. For instance, in Johns Hopkins University’s process, department chairs or deans can submit an optional letter indicating the possibility for the postdoc to be hired either within the department or within another institutional department at the end of their postdoc term. Although this letter is not a guaranteed promise of a faculty position, it strengthens the potential that the department will be approved for the postdoc position. A similar policy exists at University of Colorado Boulder and Syracuse University. At UNC Chapel Hill, departments craft postdoc job descriptions with future hiring needs in mind (EAB Global, 2017). Likewise, in UMBC’s Fellowship for Faculty Diversity, the dean evaluates whether departments will be able to hire a postdoc and forwards those applications to the Executive Committee. Alternatively, some programs are only open to departments that will have upcoming faculty positions and make clear which departments are taking postdoc applications each year, such as OSU’s Dean’s Diversity Postdoctoral Fellows and the University of Missouri’s Faculty Diversity Postdoctoral Program. The overall goal of such efforts is to create scenarios wherein the postdoc is being hired into departments that will have the ability to hire in the future and give priority to those departments.

#### Negotiating Expectations and Terms

From the outset, postdoctoral conversion programs should provide transparency around the conversion process for the benefit of all relevant parties, particularly for the fellows, mentors, and departments in which fellows are appointed. In UMBC’s Fellowship for Faculty Diversity program, the fellow’s appointment letter outlines for the fellow and host department the requirements of the position, as well as salary, funding for moving expenses, travel, office space, and research^11^. Many programs, such as University of Colorado Boulder, Northeastern University, and UMCP, likewise specify that fellows must be given office space, specific minimum start-up, or professional development funds.

The extent to which conversion is discussed and formally stated during the negotiation process is nebulous. Some of the postdoc programs reviewed specifically state what the process for conversion will be on their websites (e.g., the UC System, OSU) or as part of the hiring process (UMBC’s conversion programs) and will be discussed in Stage 4, though these seemed to be the exceptions rather than the rule. Other programs, like the AGEP PROMISE Academy, acknowledge multiple pathways to conversion, one predetermined (where the fellow is intended to be hired into the tenure track at their postdoctoral institution) and one flexible (where the fellow will be assisted in finding possible future placement within the university system). The language about the possible conversion must signal to the applicant that the tenure-track position is not guaranteed and is based on performance, while still assuring fellows that conversion is intended. We recommend that both the job advertisement and offer letter use language such as “opportunity” and “intention” (instead of a “guarantee”) to transition to a tenure-track faculty position.

Formal duties of postdocs differ from program and program. As most postdoc conversion programs are located within research-intensive institutions, the postdoc’s primary duty is to develop their independent research agenda (which contrasts with typical postdoc models wherein a postdoc works on their faculty mentor’s research). For example, in UMBC’s Fellowship for Faculty Diversity, Pre-Professoriate Program, and in the AGEP PROMISE Academy, postdocs are expected to develop and further their independent research agenda to prepare them for a tenure-track position within the institution or university system, respectively. The extent to which postdocs participate in teaching varies widely. In Carleton College’s Oden Fellowship program, teaching occupies half of the fellow’s time (two courses in the first year, three in the second year). UMBC’s fellows are required to teach one course per year (though not in the first semester of the fellowship to ensure adequate time for adjusting to a new institution and getting research off the ground). Other conversion programs leave teaching duties to the discretion of the postdoc or specifically state that teaching is not expected (e.g., UMCP’s President’s Postdoctoral Fellowship Program, Northeastern University’s STEM Future Faculty Postdoctoral Fellowship Program). No formal expectations pertaining to service, including mentoring/advising, were described within conversion programs, though it seems logical that if postdocs are in classroom roles, they may be asked to informally advise and mentor students.

### STAGE 3: Fellowship Period

Stage 3 focuses on the postdoc fellowship period and covers activities of the fellow, their faculty mentor(s), and the department into which they were hired. This stage includes onboarding, professional development, community building, and evaluation. Most postdoc conversion programs (including those located at institutions within USM) specify a two-year fellowship period, with conversion to a tenure track position taking place prior to the third year, although nationally there are some exceptions (e.g., the Heanon Wilkens Fellowship at Miami University is only one year). During this period, the fellow is onboarded, mentored, and assessed.

#### Onboarding the Fellow

The onboarding process is critical to ensure postdocs feel welcome and can successfully teach and launch their research. Hiring and onboarding a new postdoc in a conversion program is generally the purview of the department and varies considerably. To ensure quality onboarding, some more centralized programs have developed standardized onboarding experiences and/or documented expectations for departments onboarding fellows. The UMBC Fellowship for Faculty Diversity program has developed several specific onboarding practices that program leadership communicates to departments and fellows prior to the start date. These practices include written guidelines that outline the responsibilities of mentors, chairs, and fellows, and checklists that spell out the expectations from the department and the provost’s office staff (including professional development resources, office space, and pay and insurance information)^12^. Onboarding should also include a substantive review of the conversion process and criteria that was hopefully discussed prior to hire. UMBC ensures the fellow, department chair, department administrative staff, primary faculty mentor, provost office administrative staff, business office, and human resources staff all review the conversion documents in a meeting together so there are no questions left unanswered. These meetings are recorded and accessible to stakeholders at any time in the future.

Onboarding also involves creating connections between fellows and department members. UMBC’s Pre-Professoriate program in the natural sciences hires fellows on the standard academic job cycle intentionally to facilitate fellows’ ability to participate in all campus new faculty activities (orientations, socials, open houses, etc.), thereby integrating them into the department, college, and institution. UMCP’s President’s Postdoctoral Fellowship program expects fellows to participate in a program orientation and a program reception. They also provide expectations for host departments to “welcome the fellow into the department and make every effort to ensure that the fellow is included in communications about departmental colloquia, seminars and social events.” The postdoc conversion program at University of Colorado Boulder likewise specifies that departments should take action to ensure the fellow is included as a faculty member in the department. All these practices serve to establish scholars as a colleague/potential colleague and not an “inferior” trainee.

#### Fellow Professional Development

Nearly all the conversion models in our review mentioned that fellows would be invited or expected to participate in professional or career development. At the same time, there was wide variation in the extent to which these expectations were formalized and the kinds of activities in which fellows participated.

An existing best practice is the use of individual development plans (IDPs) or individual mentoring plans. IDPs serve many goals, including establishing long and short-term career goals, identifying specific activities in which the fellow will partake, marking progress over time, and structuring informal/formal evaluation of postdocs by their mentors and departments. The goal of IDPs in conversion programs should be to lay a specific professional development path that will ready the postdoc for the successful and smooth transition to a tenure-track faculty role in the department. Several USM institutions use individual development or mentoring plans in their postdoc conversion models, including the programs at UMBC, UMCP, and the AGEP PROMISE Academy. In UMBC’s Fellowship for Faculty Diversity, for instance, departments who host a postdoc create and submit “Faculty Development Plans,” which detail the research, teaching, and professional development goals for the upcoming semester. At the end of each semester, mentors and fellows submit an assessment report that reviews their progress and addresses any challenges. The AGEP PROMISE Academy has developed a standardized self-assessment tool to assist fellows and mentors identifying areas of growth and opportunity to increase chances of success securing (and being successful with) a tenure-track position^13^. Conversion programs at the University of New Hampshire and UNC Chapel Hill likewise require postdocs to create IDPs with their mentor and revisit them periodically to assess progress.

In conjunction with IDPs, most conversion programs offer or require fellows to participate in ongoing professional and career development. Professional development activities include workshops on teaching, grant-making, mentoring, or other skills development topics. Other activities might include discussions of work-life integration or maintaining productivity. The extent to which such professional development activities are offered centrally or by each postdoctoral fellows’ department varies substantially. For example, in UMBC’s Fellowship for Faculty Diversity program, professional development is largely the responsibility of the department, whereas at UMCP, the office of postdoctoral affairs offers central professional development workshops and training (in addition to any activities or workshops at the department level). In contrast, the AGEP PROMISE Academy employs a consortia model where professional development is offered to fellows across institutions, leveraging the strengths of institutions with different missions (e.g., pedagogical workshops from the teaching-centered institutions, grant writing from the medical school).

Another issue relates to the quantity and quality of professional development provided. UMBC Fellowship for Faculty Diversity program leaders noted that the kinds of activities in which postdocs participate varies widely, with some completing many and others relatively fewer. In contrast, the UMBC Pre-Professoriate program specifies at least three, institution-level professional activities fellows are expected to complete at minimum (a 4-day entrepreneurship training program, a STEM teaching series that leads to an internally recognized certificate, and an inter-department mentoring program). In the latter case, the Pre-Professoriate program integrated existing campus and unit-level faculty development activities into the requirements for fellows.

There are benefits and limitations of any approach. Department-level professional development potentially provides postdocs with local and discipline-specific knowledge that the postdoc can then leverage as a faculty member. Institutional and cross-institutional programs provide opportunities for networking and community building and potentially reduce program duplication but may also require more centralized coordination. The takeaways here are that program leaders might wish to establish baseline professional development expectations while still allowing for flexibility based on relevant disciplinary, institutional, and individual contexts. Moreover, leveraging existing professional development resources, at either the campus or consortia level, may be useful in areas more universal to the faculty experience (e.g., work-life integration) but less so at the disciplinary or institutional level.

#### Cohort Models

Many of the postdoc conversion programs we reviewed seemed to establish postdoc cohorts or recruit multiple fellows to begin their fellowship at the same time. For instance, the University of Illinois at Chicago’s Bridge to the Faculty (B2F) uses a cohort model to provide a community to its fellows where they participate in group meetings and workshops that build skills toward tenure track roles together. The University of Rhode Island’s Distinguished Multicultural Postdoctoral Fellows program aims to “cluster-hire” three fellows in distinct disciplines around a theme this coming year, providing offices in the same building to facilitate connection. Using a cohort model benefits the institution in that the processes of recruitment, hiring, and onboarding occur synchronously - this is especially true for programs that run in alternate years (e.g., UMBC’s Fellowship for Faculty Diversity, Carleton College’s Oden Postdoctoral Fellowship). Relatively few conversion programs reviewed distinctly named cohort building as a goal, representing a potentially untapped opportunity.

#### Fellow Evaluation, Reporting and Accountability

Many conversion programs include annual review processes. Annual reviews take two forms, though each is typically tied to the postdoc’s individual development or mentoring plans and department/mentor expectations guidelines. First, some postdoc conversion programs like UMBC’s Pre-Professoriate program and the UMCP President’s Postdoctoral Fellowship Program require that each fellow receive a formal annual review, wherein the postdoc receives feedback on their research and teaching as applicable. Likewise, postdoc programs at the University of New Hampshire and OSU specify that scholars receive an annual written performance review. These types of reviews are akin to faculty annual review processes and thus prepare them for the tenure track. Ideally, annual reviews (like IDPs) provide fellows feedback about their progress toward conversion within the department in which they are working (as opposed to more general feedback on research).

Another kind of annual review takes place at the institutional level, wherein departments, mentors, and postdocs complete assessments and submit them to central administrators. For instance, in the UMBC’s Fellowship for Faculty Diversity, program leaders established templates for annual reporting and required the postdoc and their mentor to complete the report each semester. Such reporting allows program leadership to monitor for potential issues and anticipate which departments would be hiring in the coming academic year. Reporting also held departments, mentors, and postdocs accountable for completing the activities laid out in mentoring or professional development plans, and allowed the Executive Committee to suggest and support interventions that may be deemed necessary for the fellow’s professional development.

### STAGE 4: Conversion to the Tenure Track

Conversion describes the formal transition of a postdoctoral fellow into a tenure-track faculty position, including the process and procedures for how to evaluate the fellow. As most campuses have very detailed procedures outlined in policy about faculty hiring, it is imperative that those establishing programs work with their shared governance process to determine a conversion pathway that is supported by the faculty within the department and the institution at large. For some campuses and programs, this is circumvented by having rigorous search processes for the postdoctoral fellow, aligned with typical national faculty searches, and search waiver policies that facilitate dean and/or provost hire. Although the details of the conversion process are likely of great interest to postdoctoral fellows and institutional leaders hoping to replicate these models, the processes remain obscure: of the 38 institutions reviewed, very few fully describe their conversion process on their websites. Actual procedures, criteria, and policies are frequently absent. Below we describe what we were able to garner regarding evaluation criteria, financial incentives, and search waiver policies enabling the conversion process.

#### Evaluation Criteria and Procedure

The criteria for tenure-track conversion eligibility varies across programs and detailed criteria were not easy to obtain online for most programs. Typically, programs allude to components of the evaluation process or hitting “benchmarks” that are not defined. For example, Wayne State University’s (WSU) Postdoctoral to Faculty Transition Fellowship program states that fellows who obtain external grants during their postdocs will be considered for tenure-track appointments at WSU with competitive compensation and startup packages. The program adds that “upon completion of a set of rigorous program milestones, fellows will be eligible for consideration for tenure-track faculty positions at Wayne State.” Likewise, University of Colorado Boulder notes that department chairs “should consider the fellow for faculty appointments and provide fellows with timely information regarding a future faculty appointment,” but does not specify how conversion will take place. Similarly, Carnegie Mellon University indicates that the fellowship offers “the possibility” of succeeding to a faculty position, but nothing further is specified on the website.

There are exceptions. OSU’s Dean’s Diversity Postdoctoral Fellowship program has a detailed program handbook^14^ that clearly outlines the annual expectations of fellows, the evaluation timeline, and recommendations for hire into a tenure track role. For UMBC’s Fellows for Faculty Diversity, the evaluation process is clearly outlined in the offer letter to the newly hired fellow and reiterated during onboarding meetings for the fellow, mentors, and departmental and institutional staff. In particular, the materials^15^ spell out that as early as the completion of the first semester in the role, the departmental faculty can vote to begin the process of conversion to tenure track. The evaluation of the fellow’s progress during the fellowship includes six components: (1) a presentation of research (and teaching, if appropriate), (2) a meeting with the department faculty, (3) a meeting with the department chair, (4) a meeting with the Dean, (5) a meeting with the Vice Provost for Faculty Affairs, and (6) at the conclusion of these conversion “interviews,” the department conducts a vote to recommend to the dean and provost the conversion to a tenure-track assistant professor. For UMBC’s Pre-Professoriate Fellows, the fellow prepares a dossier that is evaluated by the department faculty, who make a recommendation to the chair and dean about conversion to a tenure-track position. This process was intentionally designed to simulate the promotion and tenure process, to enhance the legitimacy of the fellow, increase department buy-in, and set fellows up to successfully move onto the tenure-track.

For state-wide systems who might want to jumpstart the process of conversion, implementation of these new faculty hiring pathways involves anticipation of critical roadblocks that might derail the conversion process. Challenges identified by the AGEP PROMISE Academy Alliance include establishing institutional commitments across participating institutions to the postdoctoral fellow after the fellowship; identifying search waiver processes that could facilitate conversion into a tenure-track roles at institutions across the university system; and developing hiring, onboarding and matchmaking processes for the fellow that increase their opportunities to build relationships with departments as a potential future faculty member.

As was mentioned in Stage 2, we recognize that in most conversion programs (institutional or system-wide), a tenure-track position is not a guarantee for the postdoctoral fellow. At the same time, establishing and providing as much detail as possible about the processes and/or criteria by which a tenure-track position may be offered would benefit applicants and likely strengthen the competition for these programs.

#### Financial Incentives

One way that institutions reduce the financial barrier of postdoc conversion programs is by linking the program to existing or new targets of opportunity hiring programs. For instance, at UNC Chapel Hill, the Office of the Executive Vice Chancellor and Provost provide a salary incentive for up to four years for faculty members who further the diversity goals of the department, which can be used to hire postdocs who participated in the President’s Postdoctoral Program. Likewise, UMCP recently made efforts to align the Presidential Postdoc Program with the newly re-established target of opportunity incentives for assistant and associate positions. Departments that host President’s Postdoctoral Fellowship postdocs can also apply for target of opportunity funds if they convert the postdoc to a tenure-track position (though this process is not automatic).

Another way institutions support the conversion of postdocs into faculty roles is by providing financial incentives. The UC System offers a centralized, institutional subsidy for universities that hire their President’s Postdoctoral Fellowship postdocs or Chancellor’s Postdoctoral Fellows into internal faculty roles at any of the system’s campuses^16^. Campuses receive a $85,000 faculty salary subsidy per year for 5 years. Based on our review, the UC model appears to be the only one financially centralized at a system level (the AGEP PROMISE Academy, while a system model, does not have centralized funding for the postdoctoral positions nor hiring incentives).

Financial incentives can also serve as an accountability mechanism to ensure that the department fulfills its obligation to provide professional development and support to *sustain* the converted fellow. For instance, UMBC’s Fellowship for Faculty Diversity specifies that faculty lines do not continue in the department if the converted fellow is not retained. If the fellow leaves the department even after conversion, the line cannot be filled by the department through a national search – the position returns to the control of the provost’s office, potentially to be used for a new Fellow for Faculty Diversity in an upcoming cycle. Such structures can incentivize departments to create a climate where scholars choose to stay and are supported in their advancement.

#### Search Waivers

Much like the processes put in place for partner hires or senior hires, institutions can put in place search waiver policies that departments can utilize or apply for when converting a postdoc into a faculty position. There are a few ways in which these search waivers apply to postdoc-faculty conversion. For example, departments applying for a postdoc as part of the UNC Chapel Hill Postdoctoral Fellowship for Faculty Diversity at UNC “pair” their postdoctoral line with incentive funding the completion of the fellowship term (EAB Global, 2017). Similar search waiver provisions exist at University of Colorado Boulder and UMCP. Search waivers are typically, though not exclusively, used in tandem with the financial incentives discussed previously. That is, if a department identifies a candidate that furthers the diversity goals of the unit, they will apply for both a search waiver and target of opportunity funding, effectively removing process-related barriers tied to an open search as well financial barriers related to funding a new faculty line. In some institutions, the conversion process is made easier if the fellow is considered an employee (rather than a trainee) because employees are given higher priority. For example, at University of Colorado Boulder, departments can access a search waiver if the candidate is already considered an employee.

For system-wide approaches, such as the UC System’s incentive model or USM’s AGEP PROMISE Academy, search waivers are a critical piece of how postdoctoral fellows can be pulled into tenure-track lines at institutions outside of where they completed their postdoctoral fellowship. The UC System has a system-wide policy explicitly outlining and encouraging hires from their diversity programs, while USM does not. Instead, USM relies on institutional policies of search waivers and target of opportunity hires for this process, though system wide language is being explored.

### STAGE 5: Ongoing, Iterative Evaluation for Program Improvement

Stage 5 emphasizes the importance of self-study. Achieving successful results requires an iterative evaluation process that is ongoing and involves both process and summative evaluations. This iterative practice allows the stakeholders to be reflective about the program and adjust rather than just give up without truly understanding where things went wrong.

#### Structured Program Evaluation and Documentation

It is imperative that the program have a plan for assessment at designated times for appropriate self-study. Program leadership should establish how data will be collected both quantitatively (e.g., number of hires, percent converting to the tenure track, percent retained and achieving tenure) and qualitatively (e.g., focus groups, meetings between program staff and mentors or departmental faculty). We found that relatively few postdoc conversion programs make public their evaluations, and those that do offer a more quantitative approach. For instance, the UC system reported in 2017 that over 90% of fellows were still in the UC system^17^ and UMBC reported that over 50% of fellows that participated in the Fellowship for Faculty Diversity have been retained. On the other hand, such data were rarely available, and we lack evidence about the experience of fellows and departments within these programs.

Evaluation should track successes, but also understand barriers and failures. It is important to consider why postdocs do not convert, for example. For each cohort of the Fellowship for Faculty Diversity, UMBC’s Executive Committee evaluated what worked and what did not and the lessons they learned. After each cohort is hired, program leaders administer a survey to stakeholders (e.g., department chairs, deans, fellows) about their experience. The Executive Committee also conducts exit interviews with any departing fellows (and indeed all departing faculty members) to understand why they were not retained and to understand aspects of department cultures that were unwelcoming and/or identify how resources could be deployed more strategically to ensure the fellow’s success. Program leaders keep detailed electronic notes to ensure lessons learned are not lost over time. In other words, UMBC has benefited from ongoing evaluation and has built in structured times to evaluate the ongoing successes and struggles of the program.

Postdoc conversion programs have the potential to also have impacts beyond the scholars who participate. For instance, the programs at UMBC have led to departments and programs rethinking their entire recruitment process, including crafting inclusive job advertisements, engaging in active recruitment and networking, creating shared evaluation metrics and application review procedures, and implementing welcoming interviewing processes and protocols. Additionally, the mentoring expectations and reports that are required have led many departments to develop more intentional and inclusive mentoring practices to support not only the fellows but also all pre-tenured faculty.

#### Continuous Program Improvement

As UMBC’s President Freeman A. Hrabowski often says, “success is never final.” Our five-stage process might be viewed as a mostly “finished product” but is the result of continuous organizational learning based on things that went wrong or turned out differently than originally planned. Many of the templates for offer letters, mentoring plans, individual development plans, and departmental readiness assessments discussed previously were generated in response to program failures. For instance, the UMBC Fellowship for Faculty Diversity developed a mechanism for ascertaining departmental commitment and readiness (through a submitted mentoring plan) as a response to early challenges with conversion of postdocs into faculty roles. The AGEP Promise Academy developed recruitment resources in response to challenges institutions faced in identifying postdocs; and we have recommended policies and practices to enhance clarity and transparency in the conversion process as the result of hiccups experienced in postdoc programs across the USM. Although our review of national postdoc conversion programs did not reveal similar program modifications in response to evaluation efforts, we suspect our experiences are not unique.

### Actionable Recommendations

In the previous section, we suggested a five-stage process for how institutions, systems, and multi-institution consortiums might develop, implement, and evaluate a postdoctoral conversion program aimed at enhancing faculty diversity. Based on these experiences, we have four recommendations that institutions, systems, and consortiums should consider before launching a postdoctoral conversion program.

### Assess Existing Faculty Diversity and Development Programs

When it comes to organizational diversity initiatives, there can be a tendency to “add” programs rather than assessing and utilizing models already in place (Chronicle of Higher Education [CHE], 2021). The success of the UMBC’s Fellowship for Faculty Diversity led to the construction of the parallel but unique Pre-Professoriate program in the life sciences, and subsequently the state-system AGEP PROMISE Academy approach, demonstrating how programs can build off each other and from the success of existing faculty diversity initiatives (e.g., UMBC’s ADVANCE Program). Program leaders engaged in iterative program improvement and learned from mistakes. Academic leaders considering such programs may likewise want to take stock of the existing diversity program landscape before launching a new postdoctoral conversion program.

### Cultivate Multi-Level Commitment of Financial and Human Resources

Financial support should be cost-shared, with support from central administration (i.e., provosts and/or deans for institutional models, state university systems for consortia approaches) as well as support from the department. Buy-in from department members can be generated through trainings that break down myths about the lack of diversity in doctorates, combat implicit racial bias as well as subfield/disciplinary bias, and engage department members in proactive faculty recruitment.

### Commit to Comprehensive Evaluation and Problem Solving After Failures

Program administrators should consider the systems that can be put in place to determine if departments are “ready” to recruit, onboard, support, mentor, and learn from postdocs from racially minoritized groups and hold them accountable for when they fail to live up to their obligations. There should be mechanisms in place to ensure that the department cannot make another bid for a postdoc until the department demonstrates growth and change in abilities to support and retain additional scholars. At the same time, departments that fail to retain postdocs may also be more invested in change and should be given opportunities to learn from their failures.

### Establish Fellows as Members of the Faculty From the Outset

Significant work must be done to establish incoming fellows as members of the faculty (or soon-to-be members of the faculty). This must be a multi-pronged approach, and should include joint classification or titling, access and invitation to faculty development centers/listservs, faculty onboarding and orientation events, ongoing professional development, and inclusion in faculty department meetings and decision-making. Several postdoc conversion models in our review mentioned their fellows be assigned a faculty office, for example, a gesture that has significant psychological impacts on the fellow and departmental faculty.

## Discussion

This paper drew from a review of 38 postdoctoral conversion programs as well as our own experiences as administrators, evaluators, and researchers of such programs. Across programs reviewed, we make the following observations. First, many (though not all) of the programs in our review are located at highly-ranked and research-intensive doctoral institutions. They are also mostly single-institution programs, not consortium-based approaches. This is perhaps unsurprising, given it is easier to implement programs within an institution rather than across them, research universities often have greater resources, and prestigious institutions often adopt similar tactics for addressing organizational problems. Faculty diversity is also a more critical challenge at some research-intensive institutions (Smith et al., 2012). However, we believe that multi-institutional collaborations are necessary for advancing faculty diversity (Griffin, 2020) and offer much promise for creating meaningful professional development and training opportunities and enhancing long-term collaborations between institutions with a variety of missions and resource levels.

On the other hand, creating a cross-institutional organizational change program requires significant effort, including creating new and sustainable leadership and communication channels; generating buy-in from system heads, administrators, department chairs, and individual faculty members; navigating disciplinary, departmental, and institutional silos; and understanding state and federal employment law (Tierney and Sallee, 2008; Thomas, 2018). This is not a process that should be undertaken without strategic calculation of readiness, resources, sustainability, and capabilities, human and financial.

A second observation is that all of the programs in this review reiterated the importance of faculty mentors, as signaled by the requirement that postdocs have an “assigned mentor.” However, research emphasizes the need for multiple mentors, including those within their department/discipline and from outside of it; and from mentors who share aspects of the identity (e.g., race) and those who do not (Griffin et al., 2020; Hsieh and Nguyen, 2020; Davis et al., 2021). We are also aware of the literature that shows that senior faculty of color tend to do the lion’s share of mentoring for early career faculty of color, because they are sought out, assigned, and/or prefer to assume those roles, representing a form of cultural taxation that may increase stress and burnout and lower retention (Zambrana, 2018). Thus, institutions and administrators should consider how postdocs can be plugged into mentoring networks. They should also take steps to ensure that senior faculty of color do not become the *de facto* mentors for all postdocs participating in such programs (e.g., by cultivating inclusive mentoring cultures and enhancing the ability of White faculty to mentor faculty of color).

One of the areas that is less represented in our findings is the critical importance of developing a sense of community and belonging for postdocs. Decades of research show that faculty of color often experience isolation, marginalization, and hostile climate in predominantly white institutions (Turner et al., 2008; Kelly and Winkle-Wagner, 2017). In addition to mentors, departments should encourage opportunities for fellows to share their scholarship, generate collaborative relationships within and outside of the department, connect to relevant affinity groups, and establish relationships with other faculty members at similar career stages (Fries-Britt and Snider, 2015; Martinez et al., 2017). Cohort approaches may in part meet some of these needs, but program designers should consider multi-pronged approaches at building community.

Finally, although our results suggest a general model by which postdoc conversion might occur, we also recognize that institutional type, culture, rankings, as well as departmental cultures and disciplinary norms (Kezar and Eckel, 2002) will no doubt shape the implementation and outcomes of a postdoc conversion program. For example, in some STEM fields, postdoctoral positions are a necessary step to the professoriate. There are therefore prevailing norms and expectations about the kinds of research a postdoctoral fellow should do and if they should be retained after completing their fellowship. On the other hand, in disciplines where postdoctoral fellows are less common, departments may need more support in terms of identifying good faculty mentors and orienting postdocs to the institution. In any of these contingencies, establishing thoughtful and comprehensive processes, from recruitment to conversion, and generating faculty buy-in is critical at the outset.

Ultimately, our results suggest that the creation of a postdoctoral conversion program aimed at increasing faculty diversity is an organizational change process (Kezar, 2001), not just a hiring initiative. There are several critical junctures at which the implementation of a postdoc conversion program requires a dramatic shift in policy and practice but also in culture, norms, and expectations. For instance, similar to the recommendations of those who have studied equity-minded change in higher education (Bensimon et al., 2016), many of the policies and practices outlined in this review require whole departments and colleges to take responsibility for the success of postdocs. Departments and their members are therefore engaged in, and accountable for, increasing diversity in their local context. We also note that aligning postdoc recruitment with tenure-track hiring may address some of the long-standing concerns about reliance on postdocs as sources of cheap and temporary labor (Jaeger and Dinin, 2017), in that conversion requires a long-term commitment from the hiring department. In all, the successful implementation and sustainability of a postdoctoral conversion program is incumbent upon changing processes and procedures, as well as pre-existing mindsets and behaviors that undercut diversity and change.

Our review suggests many areas for future examination. First and importantly, we know relatively little about the experiences of postdocs within these programs, including the factors that lead to successful (and unsuccessful) transition. Researchers may wish to understand the implementation of postdoc conversion programs using organizational change theories (e.g., Kezar, 2001) and better understand the mechanisms (e.g., search waivers) by which postdocs convert to faculty roles. Qualitative case studies that include interviews with postdocs and program administrators and examination of documents from the programs included in this review would contribute greatly in this area.

Second, relatively few programs make public the percentage of postdocs who successfully convert to tenure-track positions, which makes it difficult to ascertain the extent to which conversion programs serve their intended purpose of increasing faculty diversity, and what aspects of the programs (e.g., mentor professional development) seemed to be linked to success. We encourage researchers to consider multiple methods of studying the impact of such programs, for instance, using large historical databases (e.g., IPEDS) comparing institutions that have adopted such programs to those that have not and different kinds of implementation strategies (O’Meara et al., 2020).

Finally, researchers may also want to further examine how multi-institutional approaches to faculty diversity are influenced by system governance procedures, legal regulations, and differences in institutional policies, procedures, and cultures; as well as examine the potential benefits of localized (e.g., college or departmental) postdoc programs and/or drawbacks to centralized and/or multi-institutional approaches (e.g., duplication of professional development opportunities or conflicting mentoring guidance).

## Conclusion

At first glance, postdoctoral diversity programs with the goal of conversion may seem like yet another initiative focused solely on recruitment of underrepresented racial minorty scholars who managed to survive the rigors of graduate school. Instead, this research focuses on understanding *and changing* the institutional and systemic structures that lead to the loss of talent from minority backgrounds. Our national review of conversion programs and our own experiences at universities within the University System of Maryland suggest that to be successful, conversion models need to align recruitment practices with assessing readiness, cultivate academic leaders who are allies, develop mentors, put in place career development resources, and fundamentally shift institutional policies and practices. Deployed strategically and in a context-specific way, we see much potential in postdoctoral conversion programs for spurring institutional change and increasing the diversity of the faculty.

## Author Contributions

All authors contributed intellectually to the design of the manuscript through regular meetings and provided editing and commentary. DC conducted the research to identify diversity postdoctoral programs in the United States, performed the data analysis, did the literature review, authored the introduction and discussion and significant portions of the policies and practices section. AR authored and provided many of the examples from the UMBC Fellowship for Faculty Diversity (including specific policy examples and documents), provided historical context of program development both for the UMBC Fellowship and the AGEP PROMISE Academy. BE collected data, performed the analysis, found/organized references, and authored portions of the policies and practices section. WC-V helped with data collection, performed the data analysis, constructed the tables, and authored portions of the policies and practices section. WL and PM provided the institutional context and history about UMBC programs and authored examples from those programs. RC project managed the publication effort, collected the data, performed the analysis, and authored portions of the policies and practices section, particularly from the AGEP PROMISE Academy. All the authors have read and approved the final version of the manuscript.

## Conflict of Interest

The authors declare that the research was conducted in the absence of any commercial or financial relationships that could be construed as a potential conflict of interest.

## Publisher’s Note

All claims expressed in this article are solely those of the authors and do not necessarily represent those of their affiliated organizations, or those of the publisher, the editors and the reviewers. Any product that may be evaluated in this article, or claim that may be made by its manufacturer, is not guaranteed or endorsed by the publisher.

## Footnotes

1 By underrepresented racial minority groups, we refer to the National Science Foundation [NSF], and National Center for Science and Engineering Statistics [NCSES], 2017 specification of the three U.S. borne ethno-racial groups – African American/Black, Hispanic/Latinx, and/or Native American/Native Alaskan – that are underrepresented in science and engineering.

2 Full-time faculty data retrieved from https://ncses.nsf.gov/pubs/nsf21318#data-tables

3 The President’s Postdoctoral Fellowship Program is a national collaboration that originated with the University of California and affiliated national laboratories. The program includes UMCP, the University of Michigan, the University of Colorado, Stanford University, the California Institute of Technology, Carnegie Mellon University, the University of Minnesota, New York University, University of North Carolina at Charlotte, University of North Carolina at Chapel Hill, and Georgia Tech.

4 Cresiski, R. H., Ghent, C., Rutledge, J., Carter-Veale, W., Aumiller, J., Bertot, J., et al. (submitted). Developing a state university system model to diversify faculty in the biomedical sciences.

5 Many institutions have adopted the practice of using data to give context to applicant pools in faculty hiring. For instance, see the University of Wisconsin–Madison’s search and selection guidebook: https://wiseli.wisc.edu/wp-content/uploads/sites/662/2018/11/SearchBook_Wisc.pdf

6
https://drive.google.com/file/d/1arLAq_Ok8HX3Jiyoi6BYCHleQq4N3yvk/view

7
https://rescorp.org/news/2020/12/diversity-program-helps-postdocs-prepare-for-interviews

8
https://www.vanderbilt.edu/inclusive-excellence/academic-pathways-an-initiative-for-academic-diversity/

9
https://www.hhmi.org/sites/default/files/Educational%20Materials/Lab%20Management/entering_mentoring.pdf

10
https://www.btaa.org/leadership/pai/postdocs

11 In UMBC’s Pre-Professoriate program, the start-up funding for the expected tenure-track conversion is negotiated prior to the fellowship. For both programs, the funding for the salary of a tenure track position and all associated funds (including start up, travel) are encumbered at the faculty rate starting at the beginning of the fellowship.

12 Sample materials from the UMBC Fellows for Faculty Diversity Program can be found here: https://drive.google.com/drive/folders/10Zkgx25gyIbhoib00XiYsngyk4O6B04g?usp=sharing

13
https://thepromiseacademy.files.wordpress.com/2021/02/apaa-clo-skills-assessment-revised.pdf

14
https://ehe.osu.edu/sites/ehe.osu.edu/files/postdoctoral-fellowship-handbook.pdf

15 Sample materials from the UMBC Fellows for Faculty Diversity Program can be found here: https://drive.google.com/drive/folders/10Zkgx25gyIbhoib00XiYsngyk4O6B04g?usp=sharing

16
https://ppfp.ucop.edu/info/fellowship-recipients/hiring-incentive.html

17
https://ppfp.ucop.edu/info/documents/provost-letter-august-2018.pdf

## References

[B1] Bensimon E. M. Dowd A. C. Witham K. (2016). Five principles for enacting equity by design. *Diversity Democracy* 19:1,

[B2] Bhalla N. (2019). Strategies to improve equity in faculty hiring. *Mol. Biol. Cell* 30 2744–2749. 10.1091/mbc.e19-08-0476 31609672 PMC6789160

[B3] Chronicle of Higher Education [CHE] (2021). *Diversifying Your Campus: Key Insights and Models for Change.* Washington, DC: Chronicle of Higher Education.

[B4] Davis T. M. Jones M. K. Settles I. H. Russell P. G. (2021). Barriers to the successful mentoring of faculty of color. *J. Career Dev.* 08948453211013375.

[B5] EAB Global (2017). *Instilling Equity and Inclusion in Departmental Practice: Guiding Faculty Recruitment and Retention.* Washington, DC: EAB Global.

[B6] Eaton A. A. Saunders J. F. Jacobson R. K. West K. (2020). How gender and race stereotypes impact the advancement of scholars in STEM: professors’ biased evaluations of physics and biology post-doctoral candidates. *Sex Roles* 82 127–141. 10.1007/s11199-019-01052-w

[B7] Eisen A. Eaton D. C. (2017). A model for postdoctoral education that promotes minority and majority success in the biomedical sciences. *CBE Life Sci. Educ.* 16 1–11. 10.1187/cbe.17-03-0051 29196426 PMC5749967

[B8] Ezarik M. (2021). *More Discussion Than Action. Inside Higher Ed.* Available online at: https://www.insidehighered.com/news/2021/05/06/what-students-think-about-racial-justice-efforts-campus (accessed May 30, 2021).

[B9] Faupel-Badger J. Miklos A. (2016). *Institutional Research and Academic Career Development Awards (IRACDA)(K12) Outcomes Assessments.* Available online at: https://www.nigms.nih.gov/News/reports/Documents/IRACDA-outcomes-report.pdf (accessed May 30, 2021).

[B10] Flaherty C. (2021). *Intent to Hire. Inside Higher Ed.* Available online at: https://www.insidehighered.com/news/2021/08/23/ohio-state-takes-grow-your-own-approach-faculty-diversity (accessed September 30, 2021).

[B11] Fries-Britt S. Snider J. (2015). “Mentoring outside the line: the importance of authenticity, transparency, and vulnerability in effective mentoring relationships,” in *Mentoring as Transformative Practice: Supporting Student and Faculty Diversity, New Directions for Higher Education*, ed. Turner C. (Hoboken, NJ: Wiley), 3–11. 10.1002/he.20137

[B12] Gibbs K. D. Jr. McGready J. Griffin K. (2015). Career development among American biomedical postdocs. *CBE—Life Sci. Educ.* 14:ar44. 10.1187/cbe.15-03-0075 26582238 PMC4710405

[B13] Griffin K. A. (2020). “Institutional barriers, strategies, and benefits to increasing the representation of women and men of color in the professoriate,” in *Higher Education: Handbook of Theory and Research*, Vol. 35 ed. Perna L. W. (Berlin: Springer), 1–73. 10.1007/978-3-030-11743-6_4-1

[B14] Griffin K. A. Baker V. L. O’Meara K. (2020). “Doing, caring, and being: “Good” Mentoring and its role in the socialization of graduate students of color in STEM,” in *Socialization in Higher Education and the Early Career*, Vol. 7 eds Weidman J. DeAngelo L. (Berlin: Springer), 223–239. 10.1007/978-3-030-33350-8_13

[B15] Holtzclaw J. D. Morris L. G. Pyatt R. Giver C. S. Hoey J. Haynes J. (2005). FIRST: a model for developing new science faculty. *J. College Sci. Teach.* 34 24–29.

[B16] Hoppe T. A. Litovitz A. Willis K. A. Meseroll R. A. Perkins M. J. Hutchins A. (2019). Topic choice contributes to the lower rate of NIH awards to African-American/Black scientists. *Sci. Adv.* 5 1–12. 10.1126/sciadv.aaw7238 PMC6785250 31633016

[B17] Hsieh B. Nguyen H. T. (2020). Identity-informed mentoring to support acculturation of female faculty of color in higher education: an Asian American female mentoring relationship case study. *J. Diversity Higher Educ.* 13 169–180. 10.1037/dhe0000118

[B18] Jaeger A. J. Dinin A. J. (eds) (2017). *The Postdoc Landscape: The Invisible Scholars.* Cambridge, MA: Academic Press.

[B19] Jimenez M. F. Laverty T. M. Bombaci S. P. Wilkins K. Bennett D. E. Pejchar L. (2019). Underrepresented faculty play a disproportionate role in advancing diversity and inclusion. *Nat. Ecol. Evol.* 3 1030–1033. 10.1038/s41559-019-0911-5 31160738

[B20] Kelly B. T. Winkle-Wagner R. (2017). Finding a voice in predominantly white institutions: a longitudinal study of Black women faculty members’ journeys toward tenure. *Teachers College Record* 119 1–36.

[B21] Kezar A. (2001). *Understanding and Facilitating Organizational Change in the 21st Century: Recent Research and Conceptualizations.* Washington DC: ASHE-ERIC Higher Education Reports.

[B22] Kezar A. Eckel P. D. (2002). The effect of institutional culture on change strategies in higher education: universal principles or culturally responsive concepts? *J. Higher Educ.* 73 435–460. 10.1080/00221546.2002.11777159

[B23] Kezar A. Fries-Britt S. (2018). *Speaking with Truth and Acting with Integrity: Confronting Challenges of Campus Racial Climate. ACE.* Available online at: https://www.acenet.edu/Documents/Speaking-Truth-and-Acting-with-Integrity.pdf (accessed May 30, 2021).

[B24] Lambert W. M. Wells M. T. Cipriano M. F. Sneva J. N. Morris J. A. Golightly L. M. (2020). Research Culture: career choices of underrepresented and female postdocs in the biomedical sciences. *Elife* 9:e48774. 10.7554/eLife.48774 PMC6977964 31898935

[B25] Martinez M. A. Chang A. Welton A. D. (2017). Assistant professors of color confront the inequitable terrain of academia: a community cultural wealth perspective. *Race Ethnicity Educ.* 20 696–710. 10.1080/13613324.2016.1150826

[B26] McConnell S. C. Westerman E. L. Pierre J. F. Heckler E. J. Schwartz N. B. (2018). United States National Postdoc Survey results and the interaction of gender, career choice and mentor impact. *ELife* 7:e40189. 10.7554/eLife.40189 30561332 PMC6298783

[B27] Meyers L. C. Brown A. M. Moneta-Koehler L. Chalkley R. (2018). Survey of checkpoints along the pathway to diverse biomedical research faculty. *PLoS One* 13:e0190606. 10.1371/journal.pone.0190606 29338019 PMC5770033

[B28] Misra J. Kuvaeva A. O’Meara K. Culpepper D. K. Jaeger A. (2021). Gendered and racialized perceptions of faculty workloads. *Gender Soc.* 35 358–394. 10.1177/08912432211001387

[B29] National Science Foundation [NSF], and National Center for Science and Engineering Statistics [NCSES] (2017). *Women, Minorities, and Persons with Disabilities in Science and Engineering. Special Report NSF 17-310.* Available online at: www.nsf.gov/statistics/wmpd/ (accessed May 30, 2021).

[B30] O’Meara K. Culpepper D. Templeton L. L. (2020). Nudging toward diversity: applying behavioral design to faculty hiring. *Rev. Educ. Res.* 90 311–348. 10.3102/0034654320914742

[B31] Peek M. E. Kim K. E. Johnson J. K. Vela M. B. (2013). “URM candidates are encouraged to apply”: a national study to identify effective strategies to enhance racial and ethnic faculty diversity in academic departments of medicine. *Acad. Med.* 88 405–412. 10.1097/ACM.0b013e318280d9f9 23348090 PMC5044757

[B32] Settles I. H. Jones M. K. Buchanan N. T. Dotson K. (2020). Epistemic exclusion: scholar (ly) devaluation that marginalizes faculty of color. *J. Diversity Higher Educ. Advance Online Publication* 10.1037/dhe0000174[Epubahead of print].

[B33] Smith D. G. (2020). *Diversity’s Promise for Higher Education: Making it Work*, 3rd Edn. Baltimore, MD: JHU Press.

[B34] Smith D. G. Tovar E. García H. A. (2012). Where are they? A multilens examination of the distribution of full-time faculty by institutional type, race/ethnicity, gender, and citizenship. *New Direct. Institutional Res.* 2012 5–26. 10.1002/ir.20019

[B35] Thomas J. M. (2018). Diversity regimes and racial inequality: a case study of diversity university. *Soc. Curr.* 5 140–156. 10.1177/2329496517725335

[B36] Tierney W. G. Sallee M. W. (2008). Do organizational structures and strategies increase faculty diversity: a cultural analysis. *Am. Acad.* 4 159–184.

[B37] Turner C. S. V. González J. C. Wood J. L. (2008). Faculty of color in academe: what 20 years of literature tells us. *J. Diversity Higher Educ.* 1 139–168. 10.1037/a0012837

[B38] White-Lewis D. K. (2020). The facade of fit in faculty search processes. *J. Higher Educ.* 91 833–857. 10.1080/00221546.2020.1775058

[B39] Zambrana R. E. (2018). *Toxic Ivory Towers.* New Brunswick, NJ: Rutgers University Press. 10.36019/9780813593012

